# Would high-dose corticosteroid addition to multimodal cocktail periarticular injection contribute to prolonged pain control and better recovery following total knee arthroplasty?: study protocol for a randomized controlled trial

**DOI:** 10.1186/s13063-021-05655-1

**Published:** 2021-10-15

**Authors:** Chengfan Zhong, Rong He, Xiaomin Lu, Lilun Zhong, Ding-Kun Lin, Jun Liu, Da Guo

**Affiliations:** 1 Department of Orthopedic Surgery, People’s Hospital of Gaozhou, No. 89, Xiguan Road, Gaozhou City, 525200 Guangdong China; 2 Department of Orthopedic Surgery, Maoming People’s Hospital, No.101, Weimin Road, Maoming City, 525000 Guangdong China; 3 Clinical Medical College of Acupuncture Moxibustion and Rehabilitation, Guangzhou University of Chinese Medicine, No. 12, Jichang Road, Baiyun District, Guangzhou, 510405 Guangdong China; 4 Department of Orthopedic Surgery, The Second Affiliated Hospital, Guangzhou University of Chinese Medicine, No. 111 Dade Road, Guangzhou, 510120 Guangdong China

**Keywords:** Total knee arthroplasty, Postoperative pain control, Multimodal cocktail periarticular injection, Corticosteroids, Randomized controlled trial

## Abstract

**Background:**

Enhanced recovery following total knee arthroplasty (TKA) has been advocated to enhance postoperative recovery. Multimodal cocktail periarticular injection (MCPI) use for pain control in TKA has gained wide acceptance. MCPI-containing corticosteroids are believed to be an effective solution owing to their local anti-inflammatory effects and ability to reduce the local stress response postoperatively. However, there is conflicting evidence regarding its benefits. This trial aims to compare MCPI with a high dose of corticosteroid, normal dose of corticosteroid, and non-corticosteroid during TKA, to assess the effectiveness of MCPI containing corticosteroids in postoperative pain relief, functional improvement, rescue analgesia, and side effects and provide evidence that high-dose corticosteroids result in prolonged pain control and better recovery following TKA.

**Methods:**

This is a double-blinded, randomized, placebo-controlled study. A total of 234 patients scheduled for TKA will be recruited. During surgery, before wound closure, 80 ml of the cocktail analgesic will be injected into the muscle and joint capsule for local infiltration analgesia; the participants will be randomly assigned to three groups to receive a high dose of betamethasone MCPI (group H), normal dose of betamethasone MCPI (group N), and non-betamethasone MCPI (group C). The following indices will be recorded and analyzed: the strongest knee pain experienced during 90° flexion at 6 h, 24 h, 48 h, 72 h, 5 days, 14 days, and 30 days after surgery; 1 min walking ability; and circumference around the patella at 2, 5, 14, and 30 days after surgery; Knee Society knee score at 14 days and 30 days after surgery; C-reactive protein and blood sedimentation; blood sugar 2, 5, 14, and 30 days following surgery; rescue analgesic consumption; and adverse events. If any participant withdraws from the trial, an intention-to-treat analysis will be performed.

**Discussion:**

The results of this study will provide clinical evidence on the effectiveness of MCPI-containing corticosteroids in postoperative pain relief, functional improvement, rescue analgesia, and adverse events, as well as provide evidence on the efficacy of high-dose corticosteroids in prolonged pain control and better recovery following TKA.

**Trial registration:**

Chinese Clinical Trial Registry, ChiCTR2000038671. Registered on September 27, 2020.

**Supplementary Information:**

The online version contains supplementary material available at 10.1186/s13063-021-05655-1.

## Administrative information

Note: The numbers in curly brackets in this protocol refer to SPIRIT checklist item numbers. The order of the items has been modified to group similar items (see http://www.equator-network.org/reporting-guidelines/spirit-2013-statement-defining-standard-protocol-items-for-clinical-trials/).

**Table:** 

Title {1}	Would high-dose corticosteroid addition to multimodal cocktail periarticular injection attribute to prolonged pain control and better recovery after total knee arthroplasty?: study protocol for a randomized controlled trial.
Trial registration {2a and 2b}.	Chinese Clinical Trial Registry, ID: ChiCTR2000038671. This study has been registered on September 27, 2020.
Protocol version {3}	April 02, 2020, version 5.0
Funding {4}	The Natural Science Foundation of Guangdong Province, China. (No. 2021A1515011596)
Author details {5a}	(1) Chengfan Zhong, Department of Orthopedic Surgery, People’s Hospital of Gaozhou, No. 89, Xiguan Road, Gaozhou City, Guangdong 5252000, China (2) Rong He, Department of Orthopedic Surgery, Maoming People’s Hospital, No.101, Weimin Road, Maoming City, Guangdong, 525000, China (3) Xiaomin Lu, Clinical Medical College of Acupuncture Moxibustion and Rehabilitation, Guangzhou University of Chinese Medicine, No.12, Jichang Road, Baiyun District, Guangzhou, Guangdong, 510405, China (4) Lilun Zhong, Ding-Kun Lin, Jun Liu, Da Guo, Department of Orthopedic Surgery, The Second School of Clinic Medicine, Guangzhou University of Chinese Medicine, No. 111 Dade Road, Guangzhou, Guangdong 510120, China
Name and contact information for the trial sponsor {5b}	Not applicable. There is no sponsor for this study.
Role of sponsor {5c}	The funder has no input in the study design, protocol preparation, or future data analysis and interpretation.

## Introduction

### Background and rationale {6a}

Total knee arthroplasty (TKA) has been reported to be the most effective procedure for pain relief in patients with advanced osteoarthritis. Enhanced recovery following TKA has been advocated as an evidence-based perioperative care protocol to enhance postoperative recovery in several European countries [1–3]. The stress response following TKA is associated with postoperative nausea and vomiting [4], pain [5], fatigue [6], and decreased muscle strength [7]. Thus, decreasing the inflammatory response after surgery is important for TKA-enhanced recovery protocols. The use of multimodal cocktail periarticular injection (MCPI) for pain control following TKA has gained wide acceptance [8–10]. The agents mostly used in MCPI typically include corticosteroids, local anesthetics, opioids, and nonsteroidal anti-inflammatory drugs (NSAIDs) [11]. Corticosteroids are believed to be a key component owing to their local anti-inflammatory effects and their ability to reduce the local stress response to surgery [12]. However, there is conflicting evidence regarding its benefits. Some studies have shown that postoperative pain is improved with corticosteroids [13–16], whereas others have shown no benefit [17, 18]. The role of MCPI-containing corticosteroids in enhancing recovery following TKA remains inconclusive.

### Objectives {7}

We designed a three-arm, randomized controlled trial containing a high or normal dose of MCPI or an MCPI without corticosteroids to assess postoperative pain relief, functional improvement, rescue analgesia, C-reactive protein (CRP), blood sedimentation, blood sugar, and adverse events, thereby evaluating whether high-dose corticosteroid MCPI contributes to prolonged pain control and better recovery.

### Trial design {8}

**Fig. 1:**
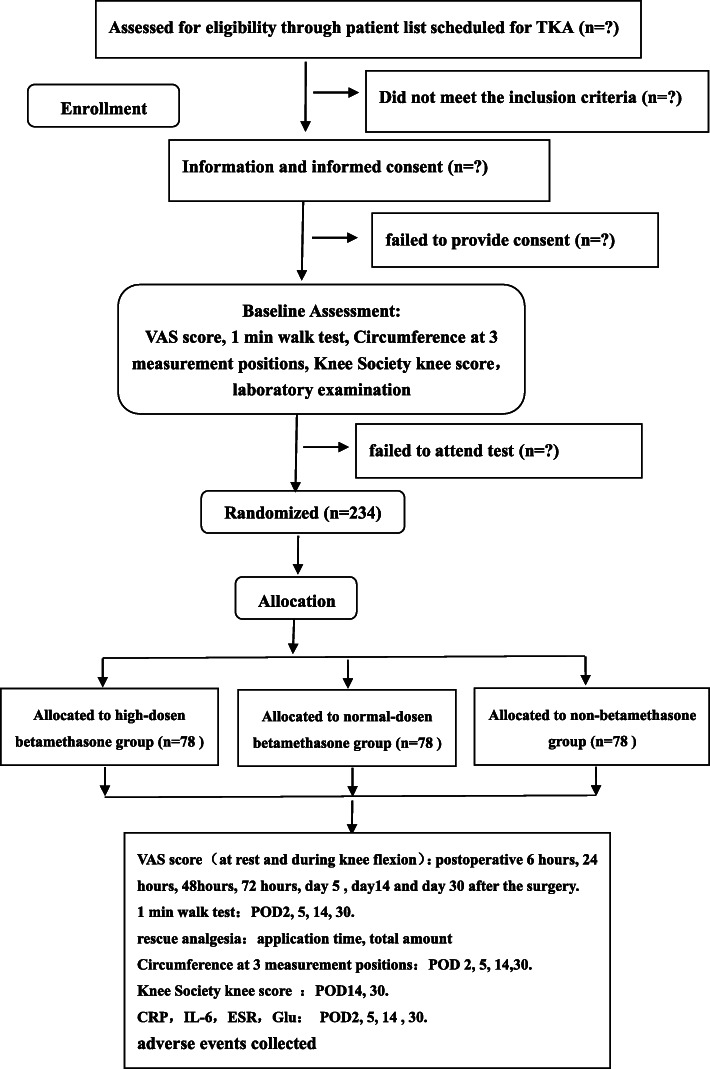
Study flowchart

This is a protocol for a three-arm, randomized, placebo-controlled trial (Fig. 1). It will follow the recommendations and suggestions of the Consolidated Standards of Reporting Trials (CONSORT 2010). The study will be conducted at a 4-week follow-up. Eligible participants will be randomized in a 1:1:1 ratio for enrollment in one of the three groups to receive a high dose of betamethasone MCPI (group H), normal dose of betamethasone MCPI (group N), and non-betamethasone MCPI (group C).

## Methods: Participants, interventions, and outcomes

### Study setting {9}

This trial has been approved by the ethics committee of The Second Affiliated Hospital of Guangzhou University of Chinese Medicine (approval number: ZF2020-031-01). Patients diagnosed with severe knee osteoarthritis (OA) and scheduled for TKA will be recruited from the Department of Orthopedics, Second Affiliated Hospital, Guangzhou University of Chinese Medicine.

### Eligibility criteria {10}

Eligible patients will include those who meet all of the following inclusion criteria and do not have any of the listed exclusion criteria.

### Inclusion criteria

- Meeting the diagnostic criteria for OA
- American Society of Anesthesiologists (ASA) physical statuses I–II
- Scheduled for unilateral TKA.
- Patients aged 50–80 years.
- Willingness to provide written informed consent and to participate in and comply with the study.

### Exclusion criteria

Participants meeting one or more of the following criteria will be excluded:

- Serious mental illness, psychological problems, and inability to communicate
- Patients with cardiovascular disease, diabetes mellitus, and poor perioperative glycemic control (fasting plasma glucose > 8 mmol/L), severe renal dysfunction, severe hepatic dysfunction, history of gastric ulcer, and coagulation disorder
- Central and peripheral nervous system problems that would not allow completion of the postoperative rehabilitation scheme
- Alcohol and drug abuse
- Allergies to the ingredients of a multimodal cocktail
- Previous major knee surgery, reoperation, or trauma to the knee during the study period

### Who will take informed consent? {26a}

All candidates will undergo a standardized interview process by the investigator (RH) and receive detailed information regarding the study and treatment. The purpose, procedures, and potential risks and benefits of the study will also be explained thoroughly to the participants by RH.

### Additional consent provisions for collection and use of participant data and biological specimens {26b}

This is not applicable. We will not apply additional consent provisions for collection. If this trial supports any further analysis not described in this primary plan, researchers will need to re-obtain consent from the participants.

### Interventions

#### Explanation for the choice of comparators {6b}

The use of MCPI to control pain following TKA has gained wide acceptance [8–10]. The agents mostly used in MCPI typically include corticosteroids, local anesthetics, opioids, and nonsteroidal anti-inflammatory drugs (NSAIDs) [11]. Corticosteroids are believed to be a key component owing to their local anti-inflammatory effects and their ability to reduce the local stress response to surgery [12]. However, there is conflicting evidence regarding its benefits regarding MCPI usage. In this study, participants will be randomly assigned to one of the three groups receiving a high dose of betamethasone MCPI, normal dose of betamethasone MCPI, and non-betamethasone MCPI groups.

#### Intervention description {11a}

All the recruited patients will receive general anesthesia. Cefamandole will be administered intravenously 30 min before and after surgery. Low molecular weight heparin for thromboprophylaxis will be administered subcutaneously 8 h postoperatively and then once daily for 2 weeks. Preoperative oral doses of 400 mg celecoxib will be administered from day 1 before surgery. Postoperatively, all the patients will receive 40 mg of parecoxib sodium twice intravenously until postoperative day (POD) 3, followed by oral doses of 200 mg celecoxib every 12 h for 4 weeks. The chief investigator (DG) will perform all the operations. A Depuy Attune PS prosthesis (Attune ®; DePuy Synthes, Warsaw, IN) will be inserted via a standard medial parapatellar approach in all patients.

#### High dose of betamethasone MCPI (group H)

During surgery, before wound closure, 80 ml of cocktail analgesic will be prepared and injected into the muscle and joint capsule for local infiltration analgesia. Group H will contain 21 mg betamethasone (3 ml), 300 mg 0.75% ropivacaine (30 ml), 5 mg morphine (1 ml), 50 mg flurbiprofen axetil (5 ml), 0.4 ml 1:1000 epinephrine, and 40.6 ml saline.

#### Normal dose of betamethasone MCPI (group N)

During surgery, before wound closure, 80 ml of cocktail analgesic will be prepared and injected into the muscle and joint capsule for local infiltration analgesia. Group N will contain 7 mg betamethasone (1 ml), 300 mg 0.75% ropivacaine (30 ml), 5 mg morphine (1 ml), 50 mg flurbiprofen axetil (5 ml), 0.4 ml 1:1000 epinephrine, and 42.6 ml saline.

#### Non-betamethasone MCPI (group C)

During surgery, before wound closure, 80 ml of cocktail analgesic will be prepared and injected into the muscle and joint capsule for local infiltration analgesia. Group C will contain 300 mg 0.75% ropivacaine (30 ml), 5 mg morphine (1 ml), 50 mg flurbiprofen axetil (5 ml), 0.4 ml 1:1000 epinephrine, and 43.6 ml saline.

#### Criteria for discontinuing or modifying allocated interventions {11b}

Participants are permitted to withdraw from the study at any time without consequence. When serious adverse events are reported during the trial, either participant is able to make a withdrawal request or the chief investigator can consider to cease the trial for the participant.

#### Strategies to improve adherence to interventions {11c}

The following measures will be taken to improve adherence to the interventions and follow-up.

1. All participants will be informed of the intervention routine, potential benefits, and risks to fully understand the significance of their involvement in the study.3
2. MCPI is a routine strategy for TKA pain control whether the patient is “in” or “out ” of the trial. As the intervention will be a one-off event, once consent is obtained, the MCPI formula according to allocation will be executed.

#### Relevant concomitant care permitted or prohibited during the trial {11d}

After surgery, tramadol hydrochloride injections or sustained-release tablets will be applied for rescue analgesia if the patient experiences a knee pain visual analog scale (VAS) score ≥ 4 cm. The participants’ usage of rescue analgesics will be recorded throughout the study. No other opiate analgesia will be permitted during the trial.

#### Provisions for post-trial care {30}

As a routine follow-up plan, participants are advised for clinic follow-up visits at 2 weeks, 4 weeks, 8 weeks, and 3 months; the investigators (CZ and LZ) will contact the participants for routine clinical follow-up as post-trial care. Previous studies on the intravenous use of corticosteroids have shown that postoperative blood glucose rise, gastrointestinal ulcers, and poor wound healing could occur. In this study, the topical use of corticosteroids may have similar risks during the trial. These adverse events are included in the common adverse events of TKA and will be covered by the government health care insurance.

### Outcomes {12}

#### Primary outcome measures

The primary outcome will be knee pain during 90° flexion via VAS pain score measured by a 10-cm horizontal line scale [19]. The patients will be asked, “How much pain do you have this moment?”, the patient will then mark on the 10-cm horizontal line VAS. Zero means “absence of pain,” while 10 represents “the worst pain” VAS has been proven to be a valid and reliable outcome measure for recording pain with ICC = 0.96 to 0.98, according to a previous study [20]. The VAS score will be evaluated at preoperative day 1; postoperative 6, 24, 48, and 72 h; day 5; day 14; and day 30 following the surgery.

### Secondary outcome measures

#### Postoperative 1-min walking ability

Physical activity is an important aspect of day-to-day life, and walking capacity is a measure of exercise tolerance that requires muscle strength. Walk tests measure the distance walked over a definite time period, with greater distances indicating better performance [21, 22]. The 1-min walk test will be measured on preoperative day 1, and POD 2, 5, 14, and 30. Participants will be asked to walk as fast as possible around a circular 20-m track over a period of 1 min. Rest breaks will be allowed if required. Walking aids can be used as required. The investigators will continuously assured that the track is consequently followed.

#### Rescue analgesia

Tramadol injection or sustained-release tablets will be used as rescue analgesia whenever patients experience a VAS score ≥ 4 cm. The application time and total amount of rescue analgesia will be recorded postoperatively.

#### Other outcomes

The circumference of the knee has been shown to have acceptable reliability for the determination of gross changes in knee swelling and inflammatory status in patients post-surgery [23]. Knee swelling will be measured using the circumference of the knee at the mid-portion of the patella with the knee in full extension. Measurements will subsequently be taken 10 cm proximal to the superior pole of the patella (thigh) and 10 cm distal to the inferior pole of the patella (calf). The skin will be marked at these points, and the tape measure will be placed circumferentially around the limb. The circumference at the three measurement positions will be measured on preoperative days 1, 2, 5, 14, and 30. The Knee Society Score will be applied to evaluate knee function on preoperative days 1, and POD 14 and 30.

Perioperative changes in serum CRP, interleukin 6 (IL-6), erythrocyte sedimentation rate (ESR), and blood glucose (Glu) will be analyzed on POD 2, 5, 14, and 30.

#### Adverse event

Adverse events during the trial include serious adverse events (SAEs) and common adverse events. SAE in TKA is defined to cause serious results that required surgical treatment or intensive treatment during the first 3 months following TKA [24, 25]. Common adverse events are minor operation-related complications requiring additional care, but little threat to the patients’ life [25–28]. A brief description of adverse events is summarized in Supplementary Table 1. It should be noted that all hospital adverse events in each patient will be collected. The total occurrence rate of common adverse events will be analyzed and defined as the presence (yes) or not (no) of any event within the entire trial. For example, if a patient experienced postoperative anemia, nausea, and hypertension during hospitalization, he or she will be counted for a “yes” only once. Moreover, if a patient only experienced vomiting one time, he or she will likewise be counted for a “yes.” This will also be applied to the computation and analysis of SAE.

#### Participant timeline {13}

**Fig. 2:**
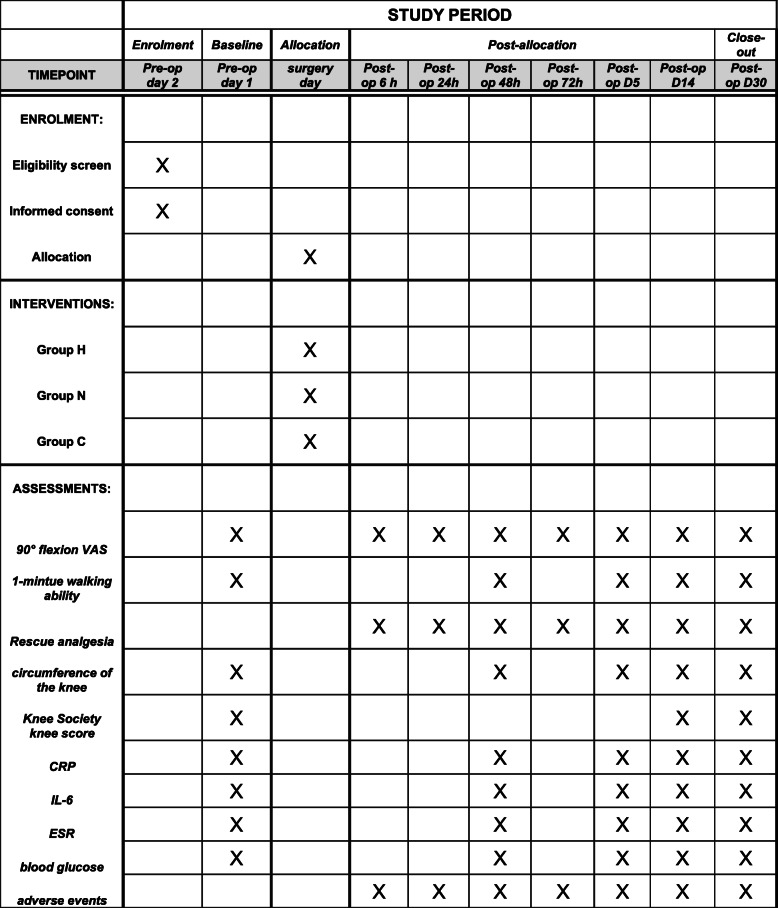
Schedule of study procedures

Figure 2 shows the time schedule of enrollment, visits for participants, intervention, and assessments.

#### Sample size {14}

The calculation of the sample size was based on previous studies. Ikeuchi et al. [13] reported that the POD 3 VAS scores were 27 ± 16 mm in the steroid group and 43 ± 18 mm in the control group. Kwon et al. [4] reported that POD 3 VAS scores were 35 mm in the steroid group and 41 mm in the control group. We assumed that the mean difference in the VAS score between the high-dose betamethasone group and control group was 0.8, and the mean difference between the normal-dose betamethasone group and control group was 0.4. An estimated standard deviation (SD) of 1.5, significance level (*α*) of 0.05, and power of test (*β*) of 0.8000 will be adopted. Based on the former data, the sample size was calculated as 69 participants per group. Assuming a dropout rate of 10%, the final sample size will be 78 participants per group, and 234 participants will be required for the three groups.

#### Recruitment {15}

Patients scheduled for TKA from the Department of Orthopedics, Second Affiliated Hospital, Guangzhou University of Chinese Medicine will be screened by the research team for participation based on the inclusion and exclusion criteria listed above. Those who satisfy the inclusion criteria and are willing to participate in this experiment will be provided details about study treatment, follow-up, and contact details for further information. Each participant will be informed of the benefits and possible risks, and they will be requested to sign the informed consent and an ethics committee-approved personal information sheet before their inclusion in the study. Recruitment began in October 2020.

### Assignment of interventions: allocation

#### Sequence generation {16a}

Random assignment will be performed by an independent investigator (DL) using a computer-generated blocked random-allocation sequence with a 1:1:1 ratio.

#### Concealment mechanism {16b}

Computer-generated numbers will be located in a sealed envelope containing the treatment information.

#### Implementation {16c}

An independent investigator (DL) will allocate the participants to the groups using the sequences generated by the computer. He will not participate in the enrollment and assessment processes. Following anesthesia, an envelope will be opened by DL and an assisting nurse who is not involved in the study, and either of them will prepare the cocktail analgesics in another room based on the assigned envelope.

### Assignment of interventions: Blinding

#### Who will be blinded {17a}

Patients will be blinded owing to general anesthesia induction. Milky white flurbiprofen axetil injection will be used as an ingredient of cocktail analgesics, which will guarantee that there are no differences in the appearance of the three groups; thus, the surgeon will be blinded. The investigators (CZ, LZ, and XL) responsible for data collection and data analysis will be blinded to the random allocation.

#### Procedure for unblinding if needed {17b}

Unblinding is permissible only when a SAE or emergency rescue occurs.

### Data collection and management

#### Plans for assessment and collection of outcomes {18a}

Before the trial, all the researchers will be trained for proficiency in the test procedures and operations. The questionnaires and laboratory tests, which will be used in this study, have been widely used in previous studies, and their reliability and validity have been reported previously.

#### Plans to promote participant retention and complete follow-up {18b}

As a routine follow-up plan, participants will be advised follow-up clinic visits at 2 weeks, 4 weeks, 8 weeks, and 3 months; investigators will contact participants for clinical follow-up.

#### Data management {19}

EpiData 3.1, software will be used for data entry. To promote the quality and accuracy of data, double data entry and checking will be managed by two data administrators (CZ and LZ). The data obtained in this study will be stored in an electronic database. The data administrators and statisticians (XL) will be the only ones with access to the information. They will also be responsible for data backup. Original case report forms and any other records will be archived at The Second Affiliated Hospital, Guangzhou University of Chinese Medicine, for a minimum of 5 years from the completion of the study.

#### Confidentiality {27}

The information collected will be used for research purposes and analyzed without the identification of the individuals involved. All personal information of the potential and enrolled participants will not be shared or released. At the end of the trial, all information will be archived at the Second Affiliated Hospital, Guangzhou University of Chinese Medicine.

#### Plans for collection, laboratory evaluation, and storage of biological specimens for genetic or molecular analysis in this trial/future use {33}

CRP, IL-6, ESR, and Glu will be measured at the corresponding time points. All specimens will be destroyed after the examinations.

### Statistical methods

#### Statistical methods for primary and secondary outcomes {20a}

Perioperative outcomes will be summarized using the mean ± SD for continuous variables and the ratio for categorical variables. All statistical analyses will be conducted using the SPSS software (version 13.0. for Windows; SPSS, Inc., Chicago, USA). For continuous variables, the Kolmogorov–Smirnov test will be used to detect the normality of distribution. Analysis of variance (ANOVA) will be used to compare the differences among the three groups, and Dunnett’s *t* test will be conducted to compare the between-group differences. For categorical variables, the chi-square test will be conducted to compare the intragroup differences, and partitions of chi-square methods will be used to compare the between-group differences.

For repeatedly measured outcomes, multivariate analysis for repeated measures ANOVA test will be performed to compare the difference between the three groups, which contains the three intervention groups, time, and group-time interaction. Dunnett’s test will be applied for post hoc comparisons. When the group-time interaction showed a significant difference, a one-way repeated measures ANOVA was conducted to identify the difference in effect between the three intervention groups. The significance level will be set at *α*=0.05. Statistical analyses will be performed by an independent investigator.

#### Interim analyses {21b}

No formal interim efficacy analyses will be conducted. Recruitment and withdrawal rates will be reviewed during the trial on an ongoing basis. The trial steering committee will review the information on the progress and accruing data of the trial (e.g., recruitment rates, data quality, adherence to protocol treatment and follow-up, main study outcomes, and safety data) and will possess the ability to propose protocol changes, modify target recruitment, and in exceptional circumstances prematurely terminate the trial.

#### Methods for additional analyses (e.g., subgroup analyses) {20b}

Not applicable. No subgroup will be established in this study.

#### Methods in analysis to handle protocol non-adherence and any statistical methods to handle missing data {20c}

An intention-to-treat analysis will be conducted according to the allocation of the original group. If any participant withdraws from the trial, the last observation carried forward method and multiple imputation method will be implemented for missing data adjustment.

#### Plans to give access to the full protocol, participant level-data and statistical code {31c}

The datasets analyzed during the current study will be available from the corresponding author upon reasonable request.

### Oversight and monitoring

#### Composition of the coordinating center and trial steering committee {5d}

The trial steering committee will be formed by the chief investigator, one investigator in this study, the coordinating center, and the administrative staff of the orthopedics department. It will be an independent group responsible for oversight of the trial and ensuring that the trial is performed according to Good Clinical Practice in order to safeguard the participants.

The coordinating center will be composed of two main investigators in this study: staff in the operating room and orthopedics clinic. They will assist with the coordination and strategic management of the trial.

#### Composition of the data monitoring committee, its role, and reporting structure {21a}

The data monitoring committee will consist of two main investigators (responsible for data administration) and staff in the ethics committee. It will be independent of the sponsor and competing interests and will be responsible for safeguarding the interests of the participants and monitoring the accumulating data.

#### Adverse event reporting and harms {22}

All SAEs will be recorded in the medical records and case report forms, including the onset date, complete description of the event, severity, duration, action taken, and outcome. The chief investigator will complete the SAE form, and the form will be emailed to the ethics committee of The Second Affiliated Hospital within five working days of awareness of the event. The chief investigator will respond to any SAE queries raised by the ethics committee as soon as possible. All common adverse events will be recorded on the case report forms.

#### Frequency and plans for auditing trial conduct {23}

The data monitoring committee will report the study progress in the form of weekly research meetings. Meanwhile, the Trial Steering Group and Ethics Committee will meet monthly to review conduct throughout the trial period.

#### Plans for communicating important protocol amendments to relevant parties (e.g., trial participants, ethical committees) {25}

Any important protocol modifications, including the principal investigator, informed consent form, study protocol regarding eligibility criteria, outcomes, and analyses, will be reported to all investigators, Trial Steering Committee, trial participants, trial registry, and ethics committee of the Second Affiliated Hospital, Guangzhou University of Chinese Medicine.

#### Dissemination plans {31a}

The results will be reported in conferences or peer-reviewed journals. There are no terms or conditions for funding that could affect publication and dissemination.

## Discussion

In TKA, an enhanced recovery strategy warrants improved patient comfort during hospital stay and accelerated discharge. Consequently, measures to control pain and accelerate rehabilitation are important. Although the MCPI technique has been proven to be a safe and cost-effective measure, the gold standard for drug combinations has not yet been established. Corticosteroid addition to MCPI is believed to be a key component owing to its local anti-inflammatory effects and ability to reduce the local stress response following surgery. However, there is conflicting evidence regarding its benefits for pain control following TKA. Christensen et al. [18] conducted a randomized controlled trial that compared patients who had MCPI with 40 mg of methylprednisolone or without corticosteroids, and the only significant result was a decreased LOS in the steroid cohort (2.6 vs. 3.5 days). There was no difference in pain scores, ROM, or Knee Society scores at any time point. Chia et al. [17] compared patients who had an MCPI containing high- or low-dose corticosteroid with patients’ MCPI without corticosteroid, and they were unable to find any significant clinical effect on postoperative ROM advantage and pain relief at the two different dosages. In contrast, Seah et al. [16], who used 40 mg of triamcinolone acetate, found a significant reduction in the early postoperative pain scores and lasting increase in the ROM in the steroid group. Wang et al. [10] found that addition of corticosteroids to an analgesic cocktail could prolong the analgesic effects of local anesthetics, reduce inflammation, slightly improve immediate postoperative pain relief, and accelerate recovery in the first 24 h following TKA. They concluded that the additional analgesic effects of corticosteroids were mild and transient, and prospective studies are still needed to determine the best cocktail of periarticular infiltration analgesia. A meta-analysis by Chai et al. [29] demonstrated that cocktail analgesia containing corticosteroid periarticular injection did not contribute to pain relief within 12 h postoperatively; however, from 24 to 72 h, it significantly decreased the pain score at rest and reduced total rescue opioids. A recent meta-analysis by Li et al. [30] demonstrated that adding corticosteroids to multimodal cocktail periarticular injection can relieve the POD 1, POD 2, and POD 3 pain intensity at rest following TKA, with no significant difference in the VAS score at the operation night, POD 4, POD 5, POD 7, and 2 weeks following the operation. Owing to these conflicting results, we plan to design a three-arm, double-blinded randomized controlled trial containing a high or normal dose of corticosteroid MCPI and a noncorticosteroid MCPI to assess their effectiveness in postoperative pain relief, functional improvement, rescue analgesia, and side effects. This study will also provide clinical evidence on the effect of high-dose corticosteroid adjuvant to MCPI on prolonged pain control and better recovery following TKA.

## Trial status

Recruitment commenced in October 2020, and the trial is scheduled to end in July 2022.

## Supplementary Information

**Additional file 1: Supplement Table 1.** Description of adverse events.

## Data Availability

The datasets used and/or analyzed during the current study will be available from the corresponding author upon reasonable request.

## Abbreviations

TKA: Total knee arthroplasty
MCPI: Multimodal cocktail periarticular injection
POD: Postoperative day
OA: Knee osteoarthritis
VAS: Visual analog scale
ROM: Range of motion
CRP: C-reactive protein
ESR: Erythrocyte sedimentation rate
IL-6: Interleukin 6
Glu: Blood glucose
SAE: Serious adverse events
