# Supplementary material for: Would high-dose corticosteroid addition to multimodal cocktail periarticular injection contribute to prolonged pain control and better recovery following total knee arthroplasty?: study protocol for a randomized controlled trial
Source: Trials. 2021 Oct 15;22:703. doi: 10.1186/s13063-021-05655-1 (PMC8518286; doi:10.1186/s13063-021-05655-1)
Supplement: Supplementary file 1 — Additional file 1: Supplement Table 1. Description of adverse events. [file 13063_2021_5655_MOESM1_ESM.docx]

**Supplement table 1.** Description of adverse events

| Adverse event | Brief description |
| --- | --- |
| **Serious adverse events:** |  |
| Tachyarrhythmia | An excessively rapid heartbeat accompanied by arrhythmia in a rate over 100 beats/min |
| Cerebrovascular accident | a sudden interruption of the blood supply to the brain caused by rupture of an artery in the brain |
| Anaphylactic shock | a severe reaction to a substance to which a person has an extreme sensitivity, may involving respiratory difficulty and circulation failure |
| Deep wound infection | prosthetic joint infection |
| Returning to the operation room | Return to the operation room for any reasons |
| Others |  |
| **Common adverse events：** |  |
| Anemia | Postoperative anemia(Hb<110 for female; Hb<120g/L for male) due to blood loss requiring additional therapy |
| Transfusion | Postoperative anemia due to blood loss requiring transfusion |
| Nausea | Nausea |
| Vomiting | Nausea with vomiting |
| Dizziness | Feel dizziness requiring taking oxygen therapy or other treatment |
| Hypertension | Systolic blood pressure≥180mmHg or diastolic blood pressure≥100mmHg requiring acute antihypertensive drugs |
| Electrolyte imbalance | Hypokalemia, hyperkalemia, hyponatremia |
| Constipation | Having difficulty defecation and requires medication |
| Headache | Headache requiring treatment |
| Hypotension | Systolic blood pressure≤90mmHg requiring increasing blood volume |
| Low oxygen saturation | Arterial oxygen saturation below 90% requiring oxygen therapy |
| Tachycardia | Heart rate continues to exceed 100 beats/min |
| Insomnia | Insomnia requires oral sleeping pills |
| Cough | Cough caused by tracheal intubation |
| Skin blisters | Skin blisters around the surgical mouth |
| Dermatitis | Dermatitis around the surgical mouth |
| Itch of skin | Itchy skin |
| Throat discomfort | Throat discomfort due to tracheal intubation |
| Dysuresia | Difficulty urinating due to anesthesia after surgery |
| Diarrhea | Diarrhea requires therapy |
| Hypercoagulable states | Abnormal FIB(>10g/L) |
| Hematoma | Subcutaneous hematoma |
| Allergy | Skin allergies around the surgical mouth or systemic skin allergies |
| Pneumonia | Mild pneumonia |
| Hyperplastic scar | Scar hyperplasia around the surgical mouth |
| Poor appetite | Poor appetite |
| Chest tightness | Chest tightness requires oxygen therapy |
| Hyperglycemia | Postoperative blood sugar rises needing to control blood sugar treatment |
| Persistent wound drainage | Wound drainage after surgery more than 48 hours |
| Dyspnea | Dyspnea requires oxygen therapy |
| Card symptoms | Upper respiratory tract symptoms such as runny nose, sneezing, and nasal congestion |
| Cervical duct | Postoperative neck tube insertion |
| Ecchymosis | Skin ecchymosis around the surgical mouth |
| Metabolic alkalosis | Blood HCO_3-_is too high (≥27mmol/L), PaCO_2_ increases. Blood pH≥ 7.45 |
| Fever | Elevated body temperature requires medication to cool down |
| Asynodia | Difficulty erection after surgery |
| Frequent micturition | Increased frequency of urination after surgery |
| Gastric hypomotility | Functional gastric hypomotility |
| Superficial surgical site infection | Superficial surgical site infection requires anti-infective treatment |
| Sustained Wound Exudation | Increased exudate around the surgical mouth |
